# Aktuelle Diagnostik und Therapie bei Ohrmuscheldysplasien und Gehörgangsfehlbildungen: Teil 2: Übersicht der verschiedenen Therapieansätze

**DOI:** 10.1007/s00106-023-01386-8

**Published:** 2023-12-04

**Authors:** Hannes H. Brandt, Daniel Bodmer

**Affiliations:** 1 Klinische Abteilung für Hals‑, Nasen‑, Ohrenkrankheiten, Universitätsklinikum St. Pölten, Dunant-Platz 1, 3100 St. Pölten, Österreich; 2 Hals-Nasen-Ohren-Klinik, Universitätsspital Basel, Basel, 4031 Petersgraben 4, Schweiz; 3 Karl Landsteiner Privatuniversität für Gesundheitswissenschaften, Krems, 3500 Dr. Karl-Dorrek-Straße 30, Österreich

**Keywords:** Kongenitale Anomalien, Rehabilitation, Cholesteatom, Hirnstammaudiometrie, Implantierbare Hörsysteme, Congenital abnormalities, Rehabilitation, Cholesteatoma, Evoked response audiometry, Implantable hearing aids

## Abstract

Angeborene Fehlbildungen der Ohrmuschel und des äußeren Gehörgangs können mit schweren ästhetischen und funktionellen Defiziten einhergehen. Neben dem Verständnis embryologischer Grundlagen ist auch die Klassifikation derartiger Anomalien für die Behandlung essenziell. Die frühzeitige Erkennung einer Fehlbildung sowie die Einleitung zeitgerechter Diagnostik sind essenziell, um durch eine adäquate Therapie langfristige Einschränkungen zu verhindern. Ohrmuscheldysplasien werden heute meist operativ, seltener mittels Epithese korrigiert. Die Methoden des „tissue engineering“ sind seit Langem in der Erprobung und könnten in Zukunft eine wesentliche Rolle spielen. Die Behandlung von Gehörgangsstenosen und -atresien bezweckt neben einer Hörverbesserung auch die Verhinderung von Folgeerkrankungen. Darunter finden sich u. a. Cholesteatome oder rezidivierende Infekte. Die Hörrehabilitation umfasst konventionelle und implantierbare Hörgeräte, wobei der Trend zu Letzteren tendiert.

## Abstract (en)

Congenital malformations of the pinna and aural atresia can result in major aesthetic and functional deficits. Knowledge about embryologic developments and established classification systems is an essential requirement when dealing with affected patients. Early detection of deficiencies and introduction of appropriate diagnostic measures is vital to initiate adequate therapies and prevent long-term disabilities. Treatment for malformations of the pinna—if requested—is mostly surgical, infrequently an epithesis is applied. As in other surgical fields, tissue engineering will likely play a crucial role in the future. Treatment of aural stenosis and atresia aims at improvement of hearing levels and prevention of secondary complications like cholesteatoma and chronic otorrhea. Auditory rehabilitation comprises a spectrum from conventional hearing aids to invasive hearing implants, the latter being favored in recent years.

## Lernziele

Nach der Lektüre dieses Beitrags…

- kennen Sie die für die verschiedenen Formen der Ohrmuscheldysplasie zur Verfügung stehenden Therapieansätze,
- überblicken Sie die im Fall einer Gehörgangsatresie möglichen, indizierten und nicht indizierten Therapien,
- verstehen Sie, wie sich die Therapien in den letzten Jahrzehnten gewandelt haben und vermutlich weiter verändern werden,
- können Sie Eltern über die zur Verfügung stehenden Therapien und deren Vor- und Nachteile aufklären.

## Einführung

Eine gründlich durchgeführte Diagnostik im Rahmen einer Ohrmuscheldysplasie oder Gehörgangsatresie ist die Basis für eine adäquate Therapie. Die operativen und konservativen Behandlungen müssen für ein gutes Ergebnis ideal aufeinander abgestimmt sein. Nachdem im ersten Teil des Beitrags Grundlagen und Diagnostik abgehandelt wurden, soll nun auf die Therapiemöglichkeiten eingegangen werden.

## Hörrehabilitation

Die wichtigste therapeutische Maßnahme im Fall einer Hörminderung ist die **frühzeitige Rehabilitation**

Frühzeitige Rehabilitation

mit einem **abnehmbaren Knochenleitungshörgerät**

Abnehmbares Knochenleitungshörgerät

(z. B. Stirnband-BAHA, „bone-anchored hearing aid“). Nur so können Entwicklungsrückstände vermieden werden. In der Literatur herrscht Uneinigkeit darüber, ob leichtgradige, einseitige Hörminderungen ebenfalls rehabilitiert werden sollten. Manche Autoren plädieren dafür, die kindliche Akzeptanz und die elterliche Meinung in der Entscheidung zu berücksichtigen [1]. Eine Übersichtsarbeit aus dem Jahr 2013 unterstützt eine Einordnung von einseitigen Schwerhörigkeiten als „medizinisch beeinträchtigt“ und befürwortet explizit eine Rehabilitation einseitiger Hörminderungen [2].

### Merke

Eine zeitnahe Rehabilitation des Hörvermögens ist sehr wichtig. Bei einem normal hörenden kontralateralen Ohr wird deren Notwendigkeit z. T. kontrovers diskutiert.

Auch wenn Kinder mit einer unilateralen Schallleitungsschwerhörigkeit auf dem Boden einer Gehörgangsatresie im Vergleich zu solchen mit einer unilateralen sensorineuralen Schwerhörigkeit bessere schulische Leistungen zu zeigen scheinen, sollte insbesondere bei **höhergradigen Schallleitungsstörungen**

Höhergradige Schallleitungsstörungen

unbedingt eine Rehabilitation empfohlen werden [3, 4].

Das Risiko einer **Sprachentwicklungsverzögerung**

Sprachentwicklungsverzögerung

bei beidseitiger Atresie bzw. beidseitiger hochgradiger Hörminderung ist deutlich höher, weshalb eine Versorgung hier bereits in den ersten Lebenswochen erfolgen sollte. Je nach Quelle kann eine Versorgung bei einseitiger Schwerhörigkeit bis ins 2. Lebensjahr warten [5].

### Merke

Der Zeitpunkt der Rehabilitation unterscheidet sich bei unilateralen und bilateralen pathologischen Veränderungen.

## Therapie der Ohrmuscheldysplasie

Gemäß internationalem Positionspapier werden bei der Therapie dysplastischer Ohrmuscheln 4 Herangehensweisen unterschieden [6]:

- keine Therapie/Rekonstruktion,
- Rekonstruktion durch autologe Knorpeltransplantation (Rippenknorpel),
- Rekonstruktion durch Polyethylen-Implantate mit oder ohne zusätzliche Deckung durch einen Faszienlappen sowie eine Deckung mit Hauttransplantat,
- Versorgung mit einer Prothese/Epithese.

### Fallbeispiel 1

Ein neugeborenes Mädchen wird Ihnen in der zweiten Lebenswoche zur Beurteilung über die Neonatologie angemeldet. In den ersten Lebenstagen waren „unterschiedlich konfigurierte Ohrmuscheln“ aufgefallen. Bei der klinischen Inspektion zeigt sich eine weitestgehend normal angelegte linksseitige Ohrmuschel mit allen relevanten Landmarken. Auf der Gegenseite findet sich eine kranial nicht adäquat ausgebildete Helixfalte mit einer nach dorsal und kranial **ausgezogenen Scapha**

Ausgezogene Scapha

(„Spock-Ohr“).

Sie informieren die Eltern über die Möglichkeit einer Therapie mithilfe einer Modellierungsschiene, welche jedoch zu diesem Zeitpunkt von den Eltern abgelehnt wird. Ein Jahr später wünschen die Eltern aufgrund der **persistierenden Deformität**

Persistierende Deformität

eine Korrektur. Sie erklären, dass die damals angebotene Therapieform aufgrund der nun fehlenden Formbarkeit des Ohrknorpels nicht mehr indiziert sei. Sie einigen sich auf eine klinische Kontrolle zur Evaluation einer operativen Korrektur im Verlauf.

### Nichtoperative Verfahren

Ausgewählte Dysplasien (z. B. leichte Formen der Tassenohrdeformität) können in den ersten Wochen nach der Geburt z. T. durch **Modellierungstechniken**

**Figure 1:**
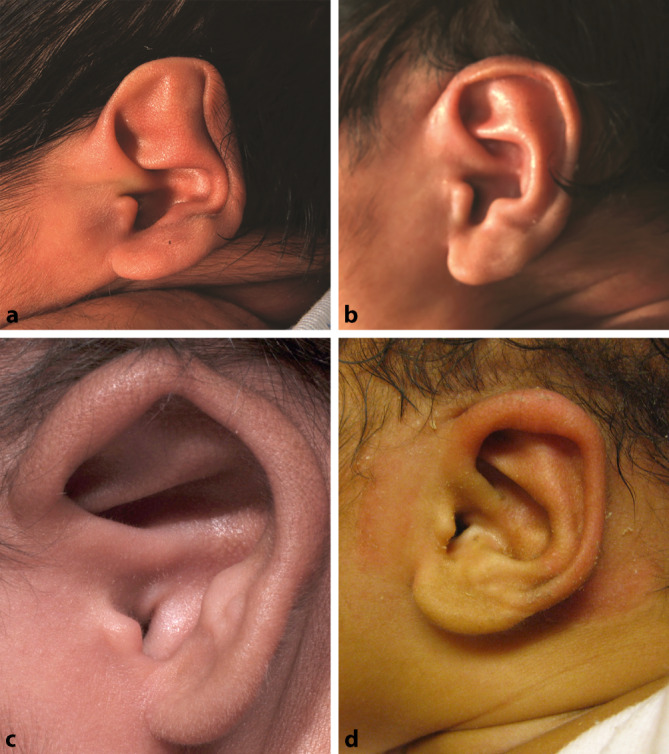

Modellierungstechniken

nichtinvasiv therapiert werden. Dadurch kann eine chirurgische Therapie in Allgemeinanästhesie mitunter vermieden werden. Während diese Methode ursprünglich nur für leichte Dysplasien angewandt wurde, können in ausgewählten Fällen auch Dysplasien mit einem gewissen Defizit an Haut und Knorpel therapiert werden. Die Ergebnisse einer Therapie mit einem derartigen Modellierungssystem werden in Abb. 1 dargestellt.

Ein Therapiebeginn mehr als 3 Monate nach Geburt ist nicht erfolgversprechend, da zu diesem Zeitpunkt die **Formbarkeit**

Formbarkeit

des Ohrknorpels bereits deutlich abgenommen hat [7]. In einer neueren Untersuchung wurde gezeigt, dass ein Therapiebeginn innerhalb der ersten beiden Lebenswochen die Therapiedauer von 6–8 Wochen auf 2 Wochen verkürzen kann [8].

Für die beschriebene Behandlung erfolgt nach Analyse der vorliegenden Dysplasie eine Ausformung der dysplastischen Anteile und die Fixation unter einer **Schutzkappe**

**Figure 2:**
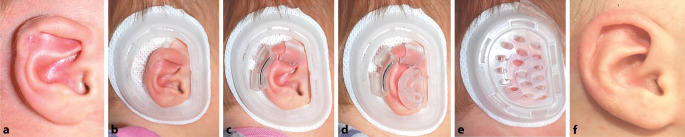

Schutzkappe

(Abb. 2). Anschließend muss das System für die gesamte Dauer der Behandlung Tag und Nacht getragen werden. In regelmäßigen Abständen sollten Wundkontrollen durchgeführt werden, da es unter der Formungsschiene zu **Druckstellen**

Druckstellen

kommen kann. Die definitive Tragedauer hängt vom Beginn der Anwendung ab. Eine neuere Publikation verspricht durch Verwendung konventioneller Materialien ein kosteneffektives Vorgehen für manche Dysplasien [9].

#### Merke

Die Korrektur leichtgradiger Dysplasien der Ohrmuschel kann, frühzeitig erkannt, in manchen Fällen durch Modellierung korrigiert werden.

### Apostasis otis

Die Mehrzahl der Ohrmuscheldysplasien sind **„Abstehohren“**

„Abstehohren“.

Sie werden ab einem bestimmten Abstehwinkel und Abstand der Concha zum Mastoid nach Weerda als eine Ohrmuscheldysplasie Grad I klassifiziert [10]. Hierbei sind die wichtigen „Landmarken“ des Ohrreliefs identifizierbar. Zugrunde liegt der Apostasis otis häufig eine Kombination aus insuffizient ausgebildeter Anthelixfalte, Hyperplasie der Concha und einem **abstehenden Lobulus**

Abstehender Lobulus

[11]. Im Alltag werden betroffene Kinder mitunter gehänselt, was eine Indikation zur Therapie und Kostenübernahme durch die Krankenkasse darstellen kann [12].

Die Korrektur dieser Dysplasien ist seit Jahrzehnten etabliert und wurde historisch mit **Nahttechniken**

Nahttechniken

(Mustardé), **Schnitt‑/Ritztechniken**

Schnitt‑/Ritztechniken

(Stenström) oder einer Kombination dieser Vorgehensweisen (z. B. nach Converse) erreicht. Eine Korrektur kann sowohl in Lokalanästhesie als auch in Allgemeinanästhesie erfolgen. Die Nachbehandlung umfasst das Tragen eines **zirkulären Kopfverbands**

Zirkulärer Kopfverband

für etwa 2 Tage postoperativ sowie im weiteren Verlauf eines zirkulären Verbands für etwa 1–2 Wochen [11, 13].

Der ideale Zeitpunkt für die Therapie leichtgradiger Ohrmuscheldysplasien scheint vor der **Einschulung**

Einschulung

zu sein. Bei Kindern unter 5 Jahren sollte bzw. kann kein derartiger Eingriff indiziert werden. Eine „prophylaktische“ Operation ist zu vermeiden, entscheidend ist der **individuelle Behandlungswunsch**

Individueller Behandlungswunsch

aufgrund der Belastung (z. B. durch Hänseleien) im Alltag. Letzterer Umstand muss dabei explizit auch beim Kind vorliegen. Wienke fasst diese Tatsache in seiner Abhandlung der juristischen Aspekte der **Otopexie** folgendermaßen zusammen: Die Indikationsstellung […] setzt eine sehr eingehende Gesprächsführung, bestenfalls mit beiden Elternteilen, sowie ausdrücklich auch unter Beteiligung des betroffenen Kindes voraus [14].

#### Merke

Die häufigste Form der Ohrmuscheldysplasie stellt die Apostasis otis dar.

### Ohrmuschelrekonstruktion

#### Bei leichten und mittelschweren Dysplasien

Die individuellen Ausprägungen der Dysplasien gehen in der Praxis sehr stark auseinander [5, 10]. In diesen Fällen ist das operative Vorgehen wesentlich von den **morphologischen Charakteristika**

Morphologische Charakteristika

der Dysplasie abhängig. Aufgrund der weitgefächerten rekonstruktiven Optionen wird auf die weiterführende Literatur verwiesen.

**Figure 3:**
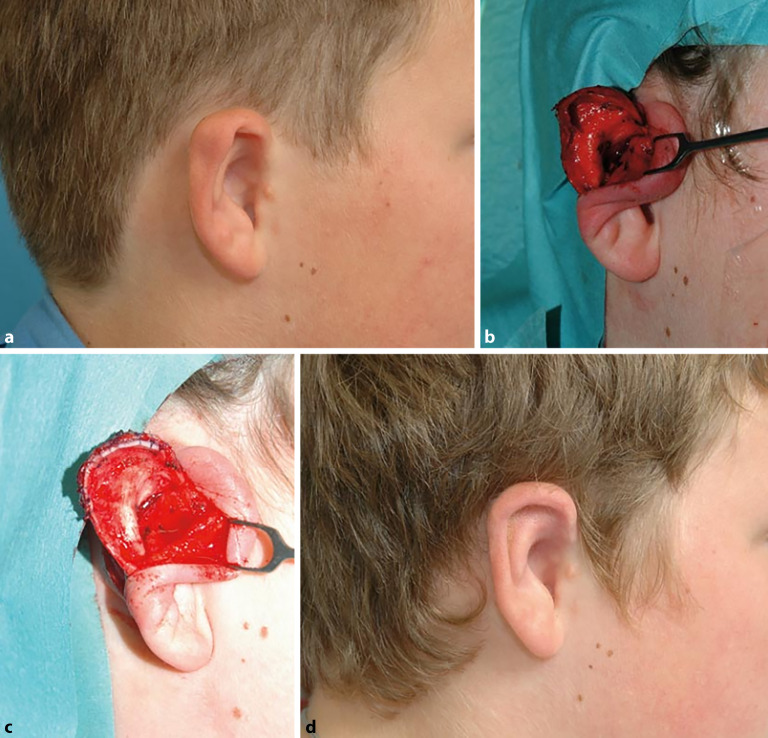

In den Abb. 3 und 4 sind 2 Beispiele entsprechender Rekonstruktion, die spezifisch für die jeweilig vorliegende Dysplasie gelten, dargestellt. Der Zeitpunkt, ab dem eine operative Intervention möglich ist, richtet sich nach dem **benötigten Knorpelvolumen**

Benötigtes Knorpelvolumen.

Wie bei der Apostasis otis muss zur Indikationsstellung ein **konkreter Leidensdruck**

Konkreter Leidensdruck

vorliegen.

**Figure 4:**
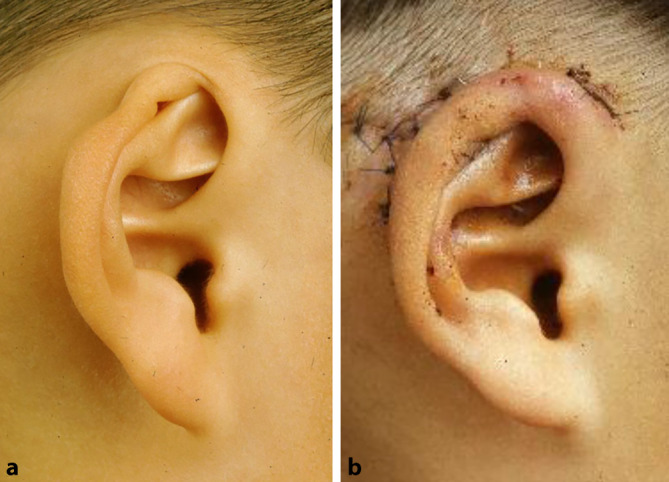

Die Kryptotie (Abb. 4) bezeichnet eine seltene Ohrmuscheldysplasie. Der kraniale Anteil der Ohrmuschel ist hier unter der Haut „vergraben“. Im asiatischen Raum tritt diese Deformität deutlich häufiger auf.

#### Bei höhergradigen Dysplasien

Fehlen manche der klassischen Landmarken einer normalen Ohrmuschel, liegt mindestens eine Ohrmuscheldysplasie Grad II vor. Hier werden für die Rekonstruktion meist zusätzliche Knorpel- und Hautanteile benötigt. Alternativ zum stützenden Knorpel können auch **Fremdmaterialien**

Fremdmaterialien,

wie Polyethylen-Netze, als Stützgerüst zum Einsatz kommen. Während Letztere einen geringeren Hebedefekt aufweisen, liegt der Vorteil der Verwendung körpereigener Transplantate in der niedrigeren Infektionsrate.

Sofern keine oder nur unzureichende Ohrmuschelanlagen ausgebildet sind, ist eine **vollständige Ohrmuschelrekonstruktion**

**Figure 5:**
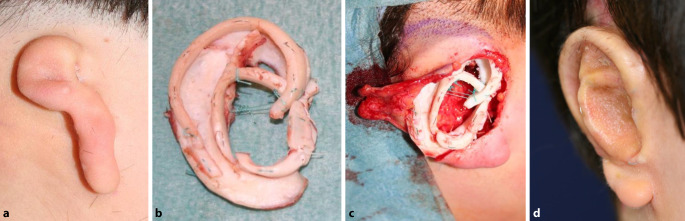

Vollständige Ohrmuschelrekonstruktion

möglich (Abb. 5). Bevor ein derartiger Eingriff indiziert wird, muss mit den Patienten und Eltern über den Grad der Beeinträchtigung, die **Erwartungshaltung**

Erwartungshaltung

an eine Rekonstruktion, den Behandlungsablauf und potenzielle Misserfolge gesprochen werden. Ein **expliziter Behandlungswunsch**

Expliziter Behandlungswunsch

nach umfassender Aufklärung hilft bei der korrekten Patientenselektion und macht eine Unzufriedenheit mit dem kosmetischen Resultat, trotz chirurgisch guter Ergebnisse, unwahrscheinlicher [17].

Für eine Rekonstruktion werden i. d. R. die synchondrotisch verbundenen Anteile der 6.–9. Rippe verwendet. Der Eingriff ist ab einem Alter von 8–10 Jahren durchzuführen [18, 19, 20]. Bei jüngeren Patienten würde sonst disproportional viel Knorpel im Vergleich zur Körpergröße entnommen, was eine ästhetisch störende Veränderung der Thoraxwand zur Folge hätte. Außerdem kann bei einseitigen Dysplasien das kontralaterale Ohr als Vorlage dienen, da es zu diesem Zeitpunkt 90 % seiner endgültigen Größe erreicht hat.

Erste publizierte Arbeiten, welche eine Rekonstruktion einer drittgradigen Ohrmuscheldysplasie aus **Rippenknorpel**

Rippenknorpel

beschreiben, stammen von Converse (1950) bzw. Tanzer (1959) [21, 22]. In den 1970er-Jahren wurde die Technik durch Brent weiterentwickelt. Er publizierte 20 Jahre später in einer Fallserie von über 500 Rekonstruktionen eine **mehrschrittige Technik**

Mehrschrittige Technik

zur Rekonstruktion der Ohrmuschel aus autologem Rippenknorpel [23]. Hierfür sind mehrere Eingriffe notwendig, die in mehrmonatigen Abständen durchgeführt werden. Die einzelnen Schritte der Rekonstruktion umfassen

- 1. Schritt: Gewinnung von Rippenknorpel und Konstruktion eines Knorpelgerüsts und Gerüstimplantation in subkutane Tasche,
- 2. Schritt: Transposition des Lobulus,
- 3. Schritt: Abheben der zunächst subkutan einliegenden Ohrmuschel von der Unterfläche,
- 4. Schritt: Rekonstruktion des Tragus.

Nagata publizierte 1994 eine andere Methode zur vollständigen Ohrmuschelrekonstruktion, welche lediglich 2 Eingriffe notwendig macht [24].

- 1. Schritt: Gewinnung von Rippenknorpel und Konstruktion eines Knorpelgerüsts sowie Insertion desselben über einen neuartigen W‑förmigen Hautschnitt in einer subkutanen Tasche,
- 2. Schritt: Anheben der rekonstruierten Ohrmuschel an ihren definitiven Ort und Rekonstruktion der retroaurikulären Hautareale mittels Vollhaut.

Bisweilen kann jedoch auch hier ein dritter Eingriff für **„Feinkorrekturen“**

„Feinkorrekturen“

notwendig werden.

Seither wurden diese Techniken weiter modifiziert, weshalb heute in verschiedenen Zentren unterschiedliche Methoden durchgeführt werden. Auch von deutschen Autoren gibt es einige umfangreiche und gut illustrierte Übersichtsarbeiten [18, 19].

##### Merke

Höhergradige Dysplasien bedürfen i. d. R. einer mehrzeitigen operativen Intervention.

### Epithesen

Eine weniger invasive Möglichkeit der Rekonstruktion ist die Anlage einer Ohrmuschelepithese. Eine Befestigung kann hierbei mittels **Hautkleber**

**Figure 6:**
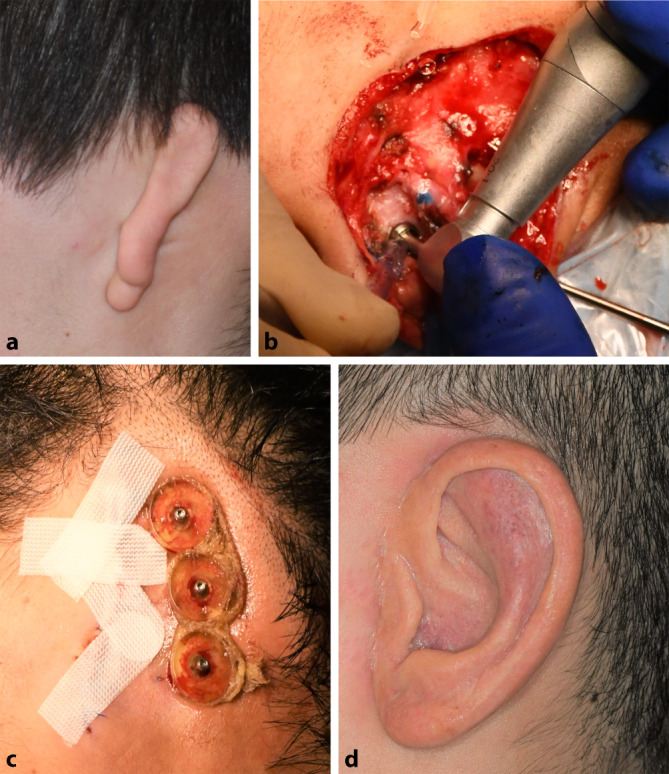

Hautkleber

oder an einer zuvor operativ eingebrachten Verankerung erfolgen (Abb. 6). Die entsprechende operative Integration der Verankerung im knöchernen Schädel wird in einer Fallserie von Somers et al. als einfach und komplikationsarm beschrieben [25]. Ein weitere Ansatz wurde von Granström publiziert – hier erfolgte die Kombination eines **knochenverankerten Hörgeräts**

Knochenverankertes Hörgerät

mit einer Epithese [26].

Erwähnenswert ist, dass Epithesen klassischerweise bei Patienten mit ungünstigen lokalen Gewebeverhältnissen (z. B. nach Bestrahlung) oder nach ausgedehnten **Tumorresektionen**

Tumorresektionen

(bessere Rezidivkontrolle) zur Anwendung kommen. Eine weitere Indikation stellt ein **fortgeschrittenes Alter**

Fortgeschrittenes Alter

dar, da hier der Rippenknorpel bereits verknöchert sein kann. Sofern für beide Möglichkeiten keine Kontraindikationen bestehen, ist die Rekonstruktion mit Rippenknorpel vorzuziehen. Diese kann bei Misserfolg oder fehlender Zufriedenheit jederzeit in eine Epithese umgewandelt werden, das Gegenteil ist selten der Fall [27].

#### Merke

Epithesen stellen neben den aufwendigen Rekonstruktionsverfahren eine etablierte Therapie dar.

### Extrakorporal gezüchtete Transplantate

Wie in anderen Bereichen der rekonstruktiven Chirurgie wird vermutlich auch in der HNO-Heilkunde das **„tissue engineering“**

„Tissue engineering“

eine Rolle spielen. Hier ist die Methode des **3‑D-Bioprinting**

3‑D-Bioprinting

erwähnenswert [28]. Ein Übersichtsartikel aus dem Jahr 2021 gibt einen Überblick der aktuell etablierten und möglicherweise in Zukunft verfügbaren Methoden [29]. Dennoch finden diese Vorgehensweise bislang noch keine breitere klinische Anwendung.

Yanaga et al. beschreiben in einer Fallserie über Patienten mit Mikrotie die erfolgreiche Anzucht einer **Knorpelmatrix** aus Chondrozyten und nachfolgender Rekonstruktion der Ohrmuschel [30]. Auch wenn diese Publikation bereits über 10 Jahre alt ist, existieren bezüglich Langzeitresultaten keinen verfügbaren Folgearbeiten. Die größte Herausforderung dieser Methode ist das Überleben des Transplantats in seiner ursprünglichen Form. Probleme sind weiter das Erreichen einer **suffizienten Vaskularisierung**

Suffiziente Vaskularisierung.

Die Erfolge aus Kleintiermodellen können bislang noch nicht in Großtierversuchen reproduziert werden [31]. Eine breite klinische Anwendung gezüchteter Transplantate scheitert aktuell noch an verschiedenen Faktoren.

#### Merke

Extrakorporal gezüchtete Transplantate werden seit Jahrzehnten im Tierversuch erprobt, aber noch nicht breit klinisch eingesetzt.

## Therapie der Gehörgangsatresie und -stenose

Eine ausführliche Übersichtsarbeit von Lo et al. beschreibt für die Therapie der Gehörgangsdysplasien (je nach Ausprägung) 4 mögliche Herangehensweisen [32].

### Keine Intervention

Die Möglichkeit, von einer weiterführenden Therapie abzusehen, besteht bei Patienten mit **einseitiger Gehörgangsatresie**

Einseitige Gehörgangsatresie

und einem normal hörenden kontralateralen Ohr. Im Abschnitt „Hörrehabilitation“ wurde bereits auf die Kontroverse hingewiesen, ob in diesem Fall bezüglich der weiteren schulischen und akademischen Entwicklung ein Nachteil besteht. Ein derartiges Prozedere sollte nach Evaluation der Bedürfnisse der Patienten sowie der Erwartungen der Eltern sorgfältig diskutiert werden.

### Externes Knochenleitungshörgerät

Ein häufig durch ein Stirnband oder mittels Adhäsionskleber befestigtes äußeres Knochenleitungshörgerät, ist eine nichtinvasive Möglichkeit der Hörrehabilitation. Sie dient als frühe oder **überbrückende Therapie**

Überbrückende Therapie,

bis eine definitive Lösung angestrebt wird.

Als dauerhafte Lösung kommen hier jedoch einige Nachteile zum Tragen. Die im Bereich des Mastoids angebrachte Hörhilfe ist deutlich sichtbar und kann daher als störend empfunden werden. **Lokale Hautirritationen**

Lokale Hautirritationen

und Schmerzen sowie die permanente Beeinträchtigung bei sportlicher Betätigung sind hier zu nennen.

### Implantierbare Hörhilfen

Die im obigen Absatz besprochenen Hörgeräte stellen daher heute v. a. Übergangslösungen dar. Sie können ab einem Alter von wenigen Monaten bis zum Alter einer definitiven Rehabilitation des Gehörs von Nutzen sein. Implantierbare Hörhilfen kommen bei beidseitigen Atresien bereits ab dem 2. Lebensjahr, bei einseitigen Atresien etwa ab dem 5. Lebensjahr zum Einsatz [6, 20]. Im Vergleich zu den im nächsten Absatz besprochenen Rekonstruktionsmaßnahmen des atretischen Gehörgangs werden diese Methoden bei der Hörrehabilitation immer mehr bevorzugt.

Eine Hörrehabilitation erfolgt somit häufig mit einem Knochenleitungshörgerät ohne Therapie der zugrunde liegenden Gehörgangspathologie. Diesbezüglich stehen **perkutane Lösungen**

Perkutane Lösungen

zur Verfügung, bei denen eine durch die Haut in die Kalotte eingebrachte Schraube als Halterung für einen Signalprozessor fungiert („bone-anchored hearing aid“, BAHA). Der hierfür notwendige Eingriff ist wenig invasiv, es besteht jedoch lebenslang eine **Kontinuitätsunterbrechung**

Kontinuitätsunterbrechung

der Kopfhaut. Das Risiko für **Infektionen**

Infektionen

und chronische Hautirritationen ist deutlich erhöht.

Eine weitere Maßnahme stellen **transkutane Knochenleitungshörgeräte**

Transkutane Knochenleitungshörgeräte

dar. Hier wird zwischen passiven und aktiven Systeme unterschieden. Bei **passiven Systemen**

Passive Systeme

werden akustische Signale außerhalb der intakten Haut in Vibrationen umgewandelt und passiv auf den Schädelknochen weitergeleitet. Im Gegensatz dazu werden bei **aktiven Systemen**

Aktive Systeme

Schallwandler und Schwingungsgeber vollständig unter der intakten Haut implantiert. Der Sprachprozessor haftet mittels eines Magneten transkutan am implantierten System. Ein entsprechendes System wurde erstmals 2011 implantiert und hat sich seither erfolgreich etabliert [33, 34]. Hier liegt ein flächiges Implantat in einer ausgebohrten Mulde der Kalotte und wird vom Prozessor über die intakte Haut angesteuert. Eine derartige Lösung erfordert jedoch eine gewisse Kalottendicke, die in den ersten Lebensjahren nicht immer vorliegt.

Eine invasivere implantierbare Hörhilfe stellt das **aktive Mittelohrimplantat**

Aktives Mittelohrimplantat

dar. Bei diesem System erfolgt die Stimulation der Cochlea über direkte Vibrationen im Bereich der Ossikel oder des runden Fensters. Das Implantat wurde Ende der 1990er-Jahre eingeführt und fand initial v. a. bei Patienten mit hochgradigen **sensorineuralen Hörminderungen**

Sensorineurale Hörminderungen

**Figure 7:**
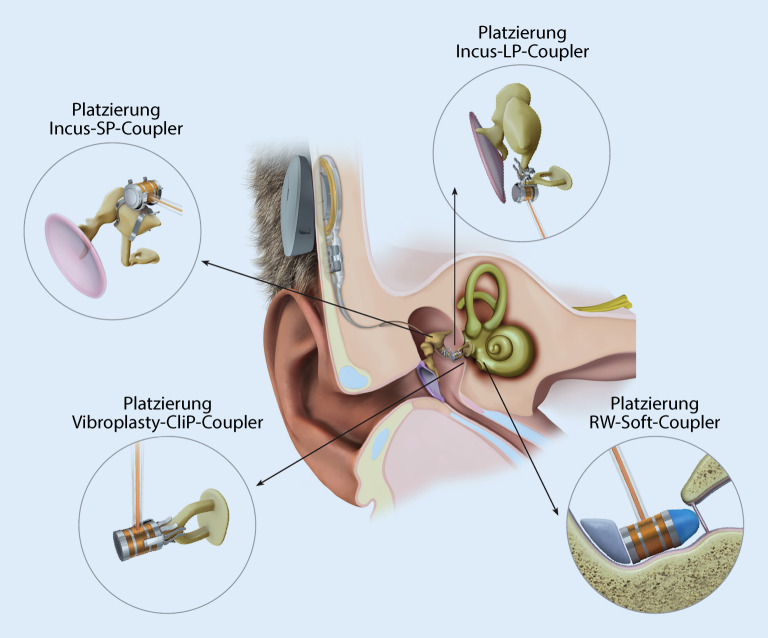

Anwendung. Ursprünglich wurde der aktive Teil (Floating Mass Transducer) nach Durchführung einer Mastoidektomie an den Incus gekoppelt. Im Verlauf wurden jedoch weitere Kopplungsmöglichkeiten eingeführt und das Spektrum so entscheidend erweitert (Abb. 7; [35]). Ein aktives Mittelohrimplantat bietet je nach anatomischer Situation verschiedene Kopplungsmöglichkeiten.

Nach der Jahrtausendwende wurde das System zunehmend auch bei **kombinierten Schwerhörigkeiten**

Kombinierte Schwerhörigkeiten

eingesetzt. Frenzel publizierte im Jahr 2009 eine Fallserie über die Anwendung bei einseitigen Gehörgangsatresien [36]. Erwähnenswert ist in diesem Zusammenhang ein von Frenzel et al. entwickelter Score. Dieser bezieht die besonderen Gegebenheiten eines dysplastischen Gehörgangs und Mittelohrs bei der Anwendung eines aktiven Mittelohrimplantats mit ein, welche durch die Scores von Jahrsdoerfer und Siegert et al. nicht adäquat abgebildet werden [37].

#### Merke

Eine Rekonstruktion bei Gehörgangsatresie aus Gründen der Hörrehabilitation erfolgt aufgrund der Fortschritte bei implantierbaren Hörgeräte seltener.

In mehreren Studien wurde eine im Vergleich zu den beiden erstgenannten Systemen bessere Hörleistung gezeigt [32]. Eine relevante Komplikation besteht in der iatrogenen Verletzung des **N. facialis**

N. facialis.

Der Nerv läuft bei Gehörgangsatresien in der Mehrzahl der Fälle weiter anterior [38]. Kiefer et al. beschreiben diesbezüglich 2 mögliche operative Zugangswege [39].

1. über eine Mastoidektomie, Antrotomie und posteriore Tympanotomie,
2. enaural über die Atresieplatte.

Im Fall einer guten Pneumatisation des Mastoids in der präoperativen Computertomographie (CT) wird Zugang 1 favorisiert, bei schlechter Pneumatisation bzw. hypoplastischem Mastoid Zugang 2.

#### Fallbeispiel 2

Eine 32-jährige Patientin gebiert in der 39. Gestationswoche nach einer weitestgehend unauffälligen Schwangerschaft ihren Sohn. Im Rahmen der initialen Beurteilung durch die Hebamme bemerkt sie eine nur rudimentär angelegte rechte Ohrmuschel auf. Nach Rücksprache mit dem zuständigen Arzt der Geburtshilfeabteilung wird der diensthabende HNO-Arzt zur Mitbeurteilung involviert. Dieser lässt das Kind wenige Tage später zur ersten klinischen Untersuchung mit anschließender Planung einer **Hirnstammaudiometrie**

Hirnstammaudiometrie

vorstellen. Letztere zeigt eine Schallleitungskomponente von etwa 50–60 dB HL. Es erfolgt die Anpassung eines abnehmbaren Knochenleitungshörgeräts.

Im Alter von 5 Jahren erhält der Patient eine hochauflösende CT des Felsenbeins, welche einen weitestgehend atretischen Gehörgang sowie **signifikante Anlagestörungen**

Signifikante Anlagestörungen

der Ossikelkette zeigt (Wert von 9 im Punktesystem zur Bewertung von Felsenbeindysplasien nach Siegert, Mayer, Weerda und Brückmann). Daher wird von einer Rekonstruktion des Gehörgangs abgeraten und die Hörrehabilitation mit einem aktiven Implantat empfohlen.

Im weiteren Verlauf erfolgt aufgrund eines entsprechenden Leidensdrucks im Alter von 9 Jahren eine mehrzeitige Ohrmuschelrekonstruktion aus Rippenknorpel.

#### Merke

Die Bandbreite an Therapien bei Gehörgangsfehlbildungen reicht vom exspektativen Vorgehen bis hin zu großen operativen Rekonstruktionseingriffen.

### Rekonstruktion

Ziel einer **Gehörgangsplastik**

Gehörgangsplastik

/„Atresioplastik“ ist das Schaffen eines trockenen und offenen Gehörgangs, um die durch den dysplastischen Gehörgang verursachte **Schallleitungsschwerhörigkeit**

Schallleitungsschwerhörigkeit

zu verbessern. Im Sprachaudiogramm werden im Mittel Schwellenwerte von 25–35 dB HL erreicht. Hierbei ist die sorgfältige Selektion geeigneter Kandidaten gemäß dem eingangs bereits beschriebenen **Jahrsdoerfer Grading System**

Jahrsdoerfer Grading System

oder der Klassifikation nach Siegert, Mayer, Weerda und Brückmann von oberster Priorität, da sich bei besonders ausgeprägten Dysplasien des Gehörgangs und des Mittelohrs keine realistische Chance auf eine postoperative Verbesserung ergibt [40]. Zur Entscheidungsfindung wird zudem die **Sprachentwicklung**

Sprachentwicklung

(sverzögerung) und die allgemeine Entwicklung des Kindes miteinbezogen.

Eine Übersicht der heutzutage angewandten Techniken geben Lee et al. [1]. **Restenosen**

Restenosen

sind mit 5–29 % eine häufige **postoperative Komplikation**

Postoperative Komplikation.

Ungefähr ein Viertel aller Patienten benötigt mindestens einen Revisionseingriff [32]. Die Rekonstruktion der Ossikelkette mit den angelegten Ossikeln scheint das Risiko eines Revisionseingriffs zu senken [41]. Ungefähr 30 % der Patienten verwenden nach einer Operation weiterhin ein Hörgerät im Alltag. Der häufig nach anterior verlagerte Verlauf des N. facialis ist auch hier relevant für eine potenzielle intraoperative Schädigung.

#### Merke

Ziel einer Gehörgangsplastik ist ein trockener Gehörgang sowie eine Verbesserung der Schallleitungsschwerhörigkeit.

Ein stenotischer Gehörgang erlaubt oft keine ausreichende Selbstreinigung, weshalb sich medial der Stenose ein **Cholesteatom**

Cholesteatom

entwickeln kann. Ist dies der Fall, besteht eine absolute Indikation zur operativen Therapie [42]. Das Risiko liegt hier gemäß einer Studie aus dem Jahr 2014 bei 20 % [43]. Patienten mit kongenitalen Gehörgangsstenosen, welche im Verlauf ein Cholesteatom entwickeln, weisen nach einer operativen Therapie **bessere Hörschwellen**

Bessere Hörschwellen

auf als jene, deren Gehörgangsstenose nicht von einem Cholesteatom verkompliziert wurde [44].

Die operative Therapie einseitiger Gehörgangsatresien kann bei normalem Hörvermögen der Gegenseite i. d. R. bis ins Jugend- oder **Erwachsenenalter**

Erwachsenenalter

verzögert werden, da hier kein Risiko einer Entwicklungsverzögerung besteht. Bei beidseitigen Gehörgangsatresien wird oft ein mehrzeitiges Vorgehen favorisiert. In der Literatur werden Rekonstruktionen üblicherweise im Alter zwischen 3 und 10 Jahren beschrieben [5, 6].

Die historische Fragestellung, welche Rekonstruktion zuerst erfolgen soll, wird mittlerweile anders beantwortet. Die meisten Autoren empfehlen zunächst eine Therapie des äußeren Ohrs, um einer Narbenbildung im rekonstruierten Gehörgang im Rahmen des Ohrmuschelaufbaus vorzubeugen. Siegert publizierte 2003 eine Methode, welche beide Rekonstruktionen kombinierte [45]. Wie jedoch erwähnt, erfolgt im Fall einer alleinigen Hörrehabilitation ohne Gehörgangsrekonstruktion die Therapie mit einem implantierbaren Hörgerät *vor *oder mit der Ohrmuschelrekonstruktion. Es sei an dieser Stelle erneut auf den bereits im ersten Teil der Fortbildung dargestellten Algorithmus verwiesen.

#### Merke

Die Aussicht auf Erfolg einer operativen Therapie kann anhand der präoperativen CT-Bildgebung abgeschätzt werden.

## Fazit für die Praxis

- Eine Rehabilitation eines möglicherweise eingeschränkten Hörvermögens ist für die normale Entwicklung betroffener Patienten die wichtigste initiale Intervention.
- Für bestimmte Ohrmuscheldeformitäten und leichtere Dysplasien existieren nichtoperative Therapien.
- Hochgradige Ohrmuscheldysplasien können operativ rekonstruiert oder mit einer Epithese versorgt werden.
- Ersterer Methode sollte der Vorzug gegeben werden.
- Das „tissue engineering“ befindet sich seit Langem in der Erprobung, kann jedoch noch nicht als relevante Alternative herangezogen werden.
- Nichtknochenverankerte Knochenleitungshörgeräte sind heute meist Übergangslösungen.
- Implantierbare Systeme stellen immer häufiger die Methode der definitiven Hörrehabilitation dar.
- Rekonstruktionsverfahren bei Dysplasien von Gehörgang und Mittelohr zur Verbesserung des Hörens werden zugunsten der implantierbaren Systeme zunehmend verlassen.
- Ob eine Rekonstruktion sinnvoll ist, hängt von der Sprachentwicklung des Kindes und der präoperativen Bewertung des Schweregrads der Dysplasie in der Feinschichtcomputertomographie des Felsenbeins ab.
- Die Eltern und, sobald möglich, auch die betroffenen Kinder sollten in die Entscheidung für oder gegen eine therapeutische Strategie miteinbezogen werden.
- Es existieren häufig mehrere Alternativen.

## CME-Fragebogen

Sie haben nach Durchführung der notwendigen Diagnostik sowie nach Diskussion der therapeutischen Möglichkeiten mit den Eltern eines 6‑jährigen Patienten mit Gehörgangsatresie und Ohrmuscheldysplasie Grad III zur Hörrehabilitation die Implantation eines aktiven Mittelohrimplantats besprochen. Welche Aussage zu diesem System ist zutreffend?

Eine Implantation benötigt i. d. R. keine Mastoidektomie.

Das Implantat wurde in den 1960er-Jahren eingeführt.

Das Fehlen des Ambosses im Mittelohr stellt eine Kontraindikation für die Implantation des genannten Hörgeräts dar.

Das Grading System nach Jahrsdoerfer ist für die Beurteilung der Durchführbarkeit einer Implantation gut geeignet.

Ursprünglich wurde das System für die Behandlung sensorineuraler Hörminderungen, erst später auch für kombinierte Hörminderungen angewendet.

Ein 10 Tage altes Neugeborenes wird von den Eltern in Ihrer Sprechstunde vorgestellt. Im Wochenbett war der Mutter ein im Seitenvergleich rechts deutlich kleineres Ohr aufgefallen. Bei Ihrer Untersuchung stellen Sie eine Ohrmuscheldysplasie Grad I nach Weerda mit einer zirkulär eingefalteten Helix und abstehendem Lobulus fest (Abb. 8). Sie denken über eine Therapie mit einem Modellierungssystem nach. Welche Aussage zur Anwendung trifft zu?

Der Patient ist zu jung für eine derartige Behandlung.

Eine Therapie mit einem Modellierungssystem dient zur Vorbereitung auf eine spätere chirurgische Intervention.

Das System sollte v. a. nachts getragen werden und kann tagsüber zur Vermeidung von Druckstellen abgenommen werden.

Die Behandlungsdauer hängt vom Alter bei Beginn der Therapie ab und kann zwischen 2 und 8 Wochen liegen.

Mit dem System können auch höhergradige Ohrmuscheldysplasien therapiert werden, wenn die Therapie frühzeitig begonnen wird.

Welche Aussage zur Therapie bei höhergradigen Ohrmuscheldysplasien trifft zu?

Eine Therapie hat nach einer eventuellen Rekonstruktion einer gleichzeitig bestehenden Gehörgangsatresie zu erfolgen.

Das „tissue engineering“ ist für die Therapie höhergradiger Dysplasien noch keine ausgereifte Methode.

Eine gleichzeitige Therapie einer knöchernen Gehörgangsatresie ist kontraindiziert.

Epithesen stellen lediglich vorübergehende Lösungen vor einer definitiven operativen Rekonstruktion dar.

Die Entnahme des Knorpels für eine Rekonstruktion erfolgt meist aus der Concha auris der Gegenseite.

Sie betreuen eine Familie, deren mittlerweile 5‑jährige Tochter an einer Anotie mit einer gleichzeitig bestehenden Gehörgangsatresie leidet. Eine computertomographische Abklärung ergab ein weitestgehend normal angelegtes Mittelohr (23 Punkte im Punktesystem zur Bewertung von Felsenbeindysplasien nach Siegert, Mayer, Weerda und Brückmann). Die Eltern wünschen eine Auskunft über den weiteren Ablauf einer Therapie. Welche Aussage ist diesbezüglich korrekt?

Der Aufbau der Ohrmuschel sollte vor der Einschulung erfolgen, um einer psychosozialen Belastung vorzubeugen.

Die operative Rekonstruktion der Gehörgangsatresie sollte im nächsten Jahr erfolgen.

Das Wachstum des kontralateralen Ohrs ist (sofern es normal ausgebildet ist) in den nächsten 6–12 Monaten abgeschlossen.

Wenn für die Rekonstruktion der Ohrmuschel autologes Material herangezogen werden soll, sind ggf. mehrere Eingriffe nötig.

Aufgrund des Werts im genannten Punktesystem sollte eine alleinige Hörgeräteversorgung und keine Rekonstruktion des Gehörgangs empfohlen werden.

Sie beraten eine Familie, bei deren 5‑jähriger Tochter klinisch eine Gehörgangsatresie rechts vorliegt. Eine Abklärung mittels Feinschichtcomputertomographie des Felsenbeins ergab normale Innenohrstrukturen, ein verlegtes Mittelohr mit fehlenden Ossikeln und verschlossenem ovalem Fenster bei einer vollständigen Atresie des rechtsseitigen Gehörgangs. Welche Aussage zur Hörgeräteversorgung trifft zu?

Das Risiko chronischer Hautirritationen und schwerer Infekte, wie beispielsweise einer Osteomyelitis, ist bei perkutanen Knochenleitungsgeräten geringer als bei transkutanen Applikationen.

Die audiometrischen Schwellenwerte eines externen Knochenleitungsgeräts sind mit denen implantierbarer Hörhilfen vergleichbar.

Beim Fehlen des Stapes, kann der sog. Floating Mass Transducer (FMT) eines aktiven Mittelohrimplantats auch direkt am runden Fenster platziert werden.

Sofern das kontralaterale Ohr der Patientin ein normales Hörvermögen besitzt, ergeben sich kaum Vorteile durch eine Versorgung mit einem implantierbaren Hörgerät.

Gemäß Jahrsdoerfer Grading System wäre eine operative Rekonstruktion des Gehörgangs und Mittelohrs eine vielversprechende Option.

Welche Aussage trifft zu implantierbaren Hörgeräten zu?

Bei vorliegender Gehörgangsatresie ist bei operativen Eingriffen auf einen aberranten Verlauf des N. facialis zu achten.

Bei aktiven Systemen liegt der Schwingungsgeber außerhalb der intakten Haut auf der Oberfläche des seitlichen Kopfs.

Aktive transkutane Knochenleitungshörgeräte können unabhängig von der Kalottendicke eingesetzt werden.

Implantierbare Hörsysteme bedürfen meist eines zweizeitigen operativen Vorgehens.

Die Implantation eines aktiven Mittelohrimplantats ist technisch weniger anspruchsvoll als die Implantation eines perkutanen Knochenleitungshörgeräts.

In Ihrer Sprechstunde stellt sich eine Familie zur Therapie einer Ohrmuscheldysplasie vor. Beim 9 Monate alten Jungen besteht eine einseitige Ohrmuscheldysplasie Grad I im Sinne einer Tassenohrdeformität Typ I. Welche Aussage zur Therapie einer Ohrmuscheldysplasie trifft zu?

Bis zum Alter von 15 Monaten können leichtgradige Deformitäten durch Modellierungstechniken konservativ therapiert werden.

Sofern eine operative Therapie gewünscht ist, wäre der ideale Zeitpunkt vor der Einschulung.

Eine Therapie im Vorschulalter ist kontraindiziert, da die Größe der Ohrmuschel im Rahmen des Wachstums bis zur Pubertät nochmals um etwa 50–70 % zunimmt.

Eine operative Therapie ist indiziert, idealerweise sollte diese nach der Pubertät geplant werden.

Hänseleien unter Kindern stellen keine Behandlungsindikation einer Ohrmuscheldysplasie dar.

Welche Aussage trifft zur Diagnostik und Therapie der Apostasis otis zu?

Bei der Apostasis otis ist die Concha auris i. d. R. hypoplastisch.

Es handelt sich um eine seltene Form der Ohrmuscheldysplasie.

Ein Teil der operativen Technik ist der Aufbau einer insuffizient ausgebildeten Anthelixfalte.

Eine konservative Therapie mit Modellierungstechniken reicht oft für ein kosmetisch zufriedenstellendes Ergebnis aus.

Die Behandlung erfolgt in Regionalanästhesie.

Für die operative Therapie einer angeborenen Gehörgangsdysplasie gilt:

Revisionsoperationen nach erfolgter Gehörgangsplastik sind häufig (etwa 25 %) und sollten im Rahmen der präoperativen Aufklärung zur Sprache kommen.

Eine potenzielle Schädigung des N. vestibulocochlearis sollte im Rahmen der Aufklärung eines Eingriffs zur Sprache kommen.

Ein niedriger Wert im Jahrsdoerfer Grading System (< 5 Punkte) spricht für ein gutes audiologisches Ergebnis nach operativer Therapie.

Bei adäquater OP-Technik ist die postoperative Schallleitungskomponente am betroffenen Ohr i. d. R. unter 10 dB HL.

Sofern die Eltern die operative Korrektur einer Gehörgangsatresie wünschen, sollte diese auch angeboten werden.

Sie planen einen Ohrmuschelaufbau bei einem Patienten mit einer angeborenen Ohrmuscheldysplasie Grad III nach Weerda. Welche Aussage zu den zur Verfügung stehenden therapeutischen Ansätzen trifft zu?

Die Operation sollte idealerweise vor dem 7. Lebensjahr erfolgen, da der Rippenknorpel dann die beste Formbarkeit für einen Ohrmuschelaufbau aufweist.

Eine Behandlung mit einer Epithese stellt bis zum Teenageralter eine gleichwertige Behandlungsmöglichkeit dar.

Auch im hohen Alter ist der Knorpel aufgrund seiner Beschaffenheit noch gut für die Rekonstruktion einer Ohrmuschel geeignet.

In der Regel wird für einen Ohrmuschelaufbau der ventrale Anteil des Knorpels der 6.–9. Rippe verwendet.

Eine Ohrmuschelrekonstruktion wird i. d. R. einzeitig durchgeführt.
